# Nitrogen trade-offs between roots and leaves of Moso bamboo and different effects of management practices on root traits and processes in subtropical forests

**DOI:** 10.3389/fpls.2025.1583127

**Published:** 2025-05-15

**Authors:** Junhui Jiang, Wenhui Shi, Yu Fu, Yuelin He, Shuyang Wang, Yeqing Ying, Lei Jiang

**Affiliations:** National Key Laboratory for Development and Utilization of Forest Food Resources, Zhejiang A&F University, Hangzhou, China

**Keywords:** Moso bamboo, root nutrient absorption, leaf nutrient resorption, root traits, different managements

## Abstract

Plant traits mediate resource acquisition strategies via trade-off between belowground root nutrient absorption and aboveground leaf nutrient resorption, yet mechanistic insights remain limited for clonal species like Moso bamboo (*Phyllostachys edulis*). This study was conducted in Moso bamboo plantations in Zhejiang Province, China. We measured rhizome-system absorptive roots, leaf properties, and soil nutrient contents to explore acquisition-resorption relationships. We also examined how management practices (abandonment [AM], conventional biennial [CM], and high-intensity annual plus understory planting [HM]) influence the traits and processes in Moso bamboo forests. Key novel findings include: (1) A consistent trade-off emerged for nitrogen [N] (negative relationships between root N absorption and leaf N resorption) but not phosphorus [P]. (2) Principal component analysis revealed root traits economics structured along two axes: first (PC1), specific root length [SRL]and root tissue density [RTD] (root lifespan) and second (PC2), cortex thickness [CT] and branching intensity [BI] (fungal independence). Interestingly, the PC1 was positively correlated with N absorption potential, and negatively correlated with N resorption efficiency. (3) HM significantly enhanced SRL (+75% vs. CM) and resorption efficiency (+23% for N, +37% for P), likely driven by interspecific competition under herb planting. While AM treatment showed relatively slight effects on traits and processes, compared with CM treatment. Our findings advance functional trait theory by decoding how clonal integration reconfigures traditional acquisition-resorption relationships, offering critical insights for bamboo forest management under global change and management.

## 1 Introduction

The root systems, hiding in the soil and directly absorbing nutrients from the soil, are crucial in carbon (C) and nutrient cycling in forest ecosystems (Ma et al., 2018; Jiang et al., 2021; Zheng et al., 2024). Recent studies of fine root systems have shown that the distal lower-order roots (i.e., order l^st^- and 2^nd^, absorptive roots) rather than higher-order roots (i.e., order 3^rd^ to 5^th^, transport roots) performed the function of absorption (Fan and Guo, 2010; McCormack et al., 2015). Moreover, the traits, such as root diameter (RD), specific root length (SRL), and root tissue density (RTD) have been verified to represent the capacity of root nutrient absorption (Bardgett et al., 2014; Bergmann et al., 2020; Zheng et al., 2024), and these traits are markedly influenced by environments and soil contexts (Gu et al., 2014; Yan et al., 2022). For example, woody trees in higher-latitude ecosystems often have narrower variations in morphological root traits (such as RD and SRL) than those in lower-latitude ecosystems to promote root resource acquisition availability (Chen et al., 2013; Gu et al., 2014). *Pinus* species had thinner absorptive fine roots and a higher SRL in young than mature trees to scavenge nutrients during stand development (Wang et al., 2024). However, such trait-environment frameworks predominantly derive from studies of non-clonal woody species, overlooking the unique rhizomatic integration in clonal plants where ramets are physiologically connected, such as Moso bamboo (*Phyllostachys edulis*) (Wang et al., 2021). This raises a critical knowledge gap: whether resource allocation trade-offs observed at the organ level in discrete-rooted trees persist in clonal systems with rhizome-system.

**Figure 1:**
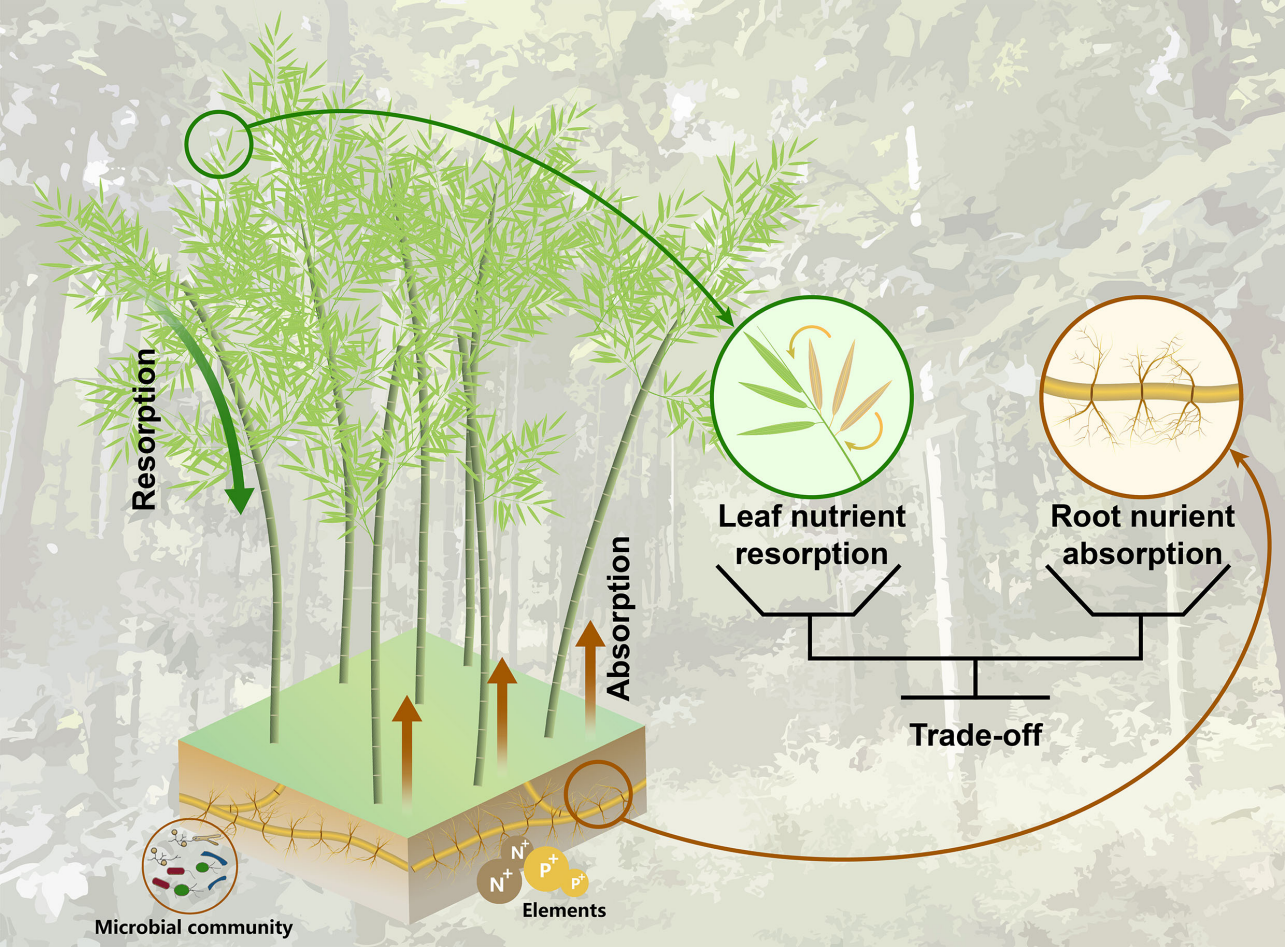
Conceptual framework illustrating the active trade-off mechanism between rhizome-system root nutrient absorption and leaf nutrient resorption in Moso bamboo (*Phyllostachys edulis*). The diagram highlights the bidirectional nutrient allocation strategies: (1) belowground nutrient acquisition via absorptive roots (1^st^ - 2^nd^ order roots) of the rhizome-system root (brown line), and (2) aboveground nutrient conservation through resorption from senescing leaves (green line). The solid black lines emphasizes the balance between root uptake and leaf uptake.

An equally important, but less well-documented resource acquisition strategy of Moso bamboo is nutrient resorption, which indicates a process by which plants withdraw nutrients from senescing tissues before abscission (Wright and Westoby, 2003). Although numerous studies have reported that nutrient resorption of woody species is an important process in explaining primary productivity in forests, annual plant demand for nitrogen (N) and phosphorus (P) is driven by N and P resorption, accounting for 30-40% at the global scale (Cleveland et al., 2013). Whether this general pattern will apply to the Moso bamboo is less clear. Recent evidence suggests that clonal integration may fundamentally alter resorption dynamics—interconnected ramets could bypass leaf senescence-related nutrient losses through internal translocation (Chen et al., 2022), theoretically negating the necessity of conventional resorption strategies. However, the trade-off between root nutrient absorption and leaf nutrient resorption for Moso bamboo has not been proven. Fortunately, our previous study addressed a plant-centred continuum, elucidating the active trade-off between root nutrient absorption from soil and nutrient resorption from senesced leaves for woody trees in subtropical forests (Jiang et al., 2023). Moso bamboo, due to its distinct physiological, structural traits such as rapid growth, and an extensive rhizome network, may allow it to simultaneously optimize both nutrient uptake and resorption from a plant-centred perspective (**Figure 1**). But, whether Moso bamboo runs this active trade-off remains limited, which creates a second knowledge gap.

Moso bamboo plays a key role in socioeconomic development in subtropical and tropical regions (Song et al., 2020), primarily due to its fast-growing (from bamboo shoots to timber approximately one and a half months), short rotation period (4 to 5 years), and explosive growth rates (Li and Feng, 2019; Yang et al., 2021). To obtain high economic benefits, bamboo forests generally receive high-intensity management, such as fertilization, reclamation, and cutting (Yang et al., 2019; Ni and Su, 2024). However, over a long-term period these management practices have resulted in negative effects, such as increased soil respiration and nutrient depletion, reduced arbuscular mycorrhizal fungal biomass, and declined stand productivity (Huang et al., 2021; Yao et al., 2022). To reduce the management cost for more profitable bamboo-related products (Cai et al., 2018), farmers have started to abandon the intense management of Moso bamboo plantations recently. This shift in management practice can significantly alter the soil properties by increasing soil organic carbon but decreasing soil water-soluble organic nitrogen (Deng et al., 2020). In addition, these changes in management practices could affect the root traits and nutrient-associated processes. For example, intensity management significantly decreased SRL and specific surface area, but increased the root biomass (Ni et al., 2021; Ni and Su, 2024), while other studies have shown the increasing nutrient resorption efficiency with bamboo expansion (Song Q. et al., 2016; Zuo et al., 2024). Despite these results, studies have rarely explored Moso bamboo rhizome-system root traits and processes synchronously, and the effects of management on them have not been tested empirically. Thus, resolving this dichotomy constitutes the third critical knowledge gap in clonal plant ecology, with in improving Moso bamboo economy and ecosystem sustainable development.

In this study, we focused on absorptive roots from the rhizome-system root subsystems of Moso bamboo species that received three different management practices, including abandonment management (no fertilization and no-tillage treatment, AM), conventional management (biennial fertilization, CM), and high-intensity management (annual fertilization plus planting with Chinese herbs under the Moso bamboo, HM) in Zhejiang Province, China. We determined the root morphological (RD, SRL, RTD, branching intensity, BI), anatomy (cortex thickness, CT), root fungal infection (mycorrhizal colonization rate, MCR), and chemical (N, P, and non-structural carbon, NSC) traits, which are closely associated with nutrient absorption and physiological metabolism, respectively. The soil properties were also measured to discuss the underlying mechanisms. Therefore, our overarching hypothesis is that (i) Moso bamboo may exhibit an active trade-off between root nutrient absorption and leaf nutrient resorption due to its unique characteristics, clonal growth; (ii) rhizome-system root traits may linked with the nutrient-associated processes, likely discovering in the woody trees; (iii) considering the strong influence of managements in Moso bamboo forests, we further speculate that compared to CM treatment, AM and HM treatment alter rhizome-system root traits and the two processes.

## 2 Materials and methods

### 2.1 Study site and field experiment

The research site was situated in Tianhuangping (30°31′20*″*N - 30°31′30*″*N, 119°35′00*″*E - 119°35′20*″*E, 200–500 m a.s.l), within Anji County, Huzhou City, Zhejiang Province, China. This region characterizes by a subtropical monsoon climate, experiences an average annual temperature of 16.1°C and receives approximately 1300 mm of precipitation annually (Ni and Su, 2024). Additionally, it enjoys an average of 1943 hours of sunshine per year and has a frost-free period spanning from 224 to 240 days. The soil at the experimental site is derived from siltstone, which is classified as red loam in the Soil Classification System (Shi et al., 2006).

Anji County is renowned globally as the birthplace of bamboo forests, encompassing roughly 1200 ha of natural Moso bamboo groves. The majority of these Moso bamboo plantations were established since 1985 with the aim of maximizing economic returns through the biennial harvesting of shoots and timber (Ni and Su, 2024). In generally, bamboo farmers take tillage and fertilization practices to management bamboo forests. Our region study included two of the most common practices, i.e., conventional management (tillage plus quadrennial fertilization, CM) and high-intensity management (tillage plus biennial fertilization plus planting of economic vegetation under the Moso bamboo forests, HM). However, due to increasing cost for more profitable bamboo-related products, farmers have started to abandon the intense management of Moso bamboo plantations recently, resulting in abandonment management (no fertilization plus no-tillage, AM). In CM and HM plantations, a blend of fertilizers consisting of urea (500 kg·ha^-1^), calcium triple superphosphate (250 kg·ha^-1^) and potassium chloride (100 kg·ha^-1^) was utilized. These fertilizers were typically spread over the soil surface after tilling the land during the autumn of a non-harvest year. In HM plantation, Chinese herbs such as *Polygonatum odoratum* (multiflowered Solomon’s seal), *Bletilla striata* (white orchid), and *Hedyotis diffusa* (three-leaf green) are cultivated under the bamboo forests. In general, bamboo trees aged five years or older are harvested for timber every two years in August, ensuring a uniform age distribution within the stand.

**Figure 2:**
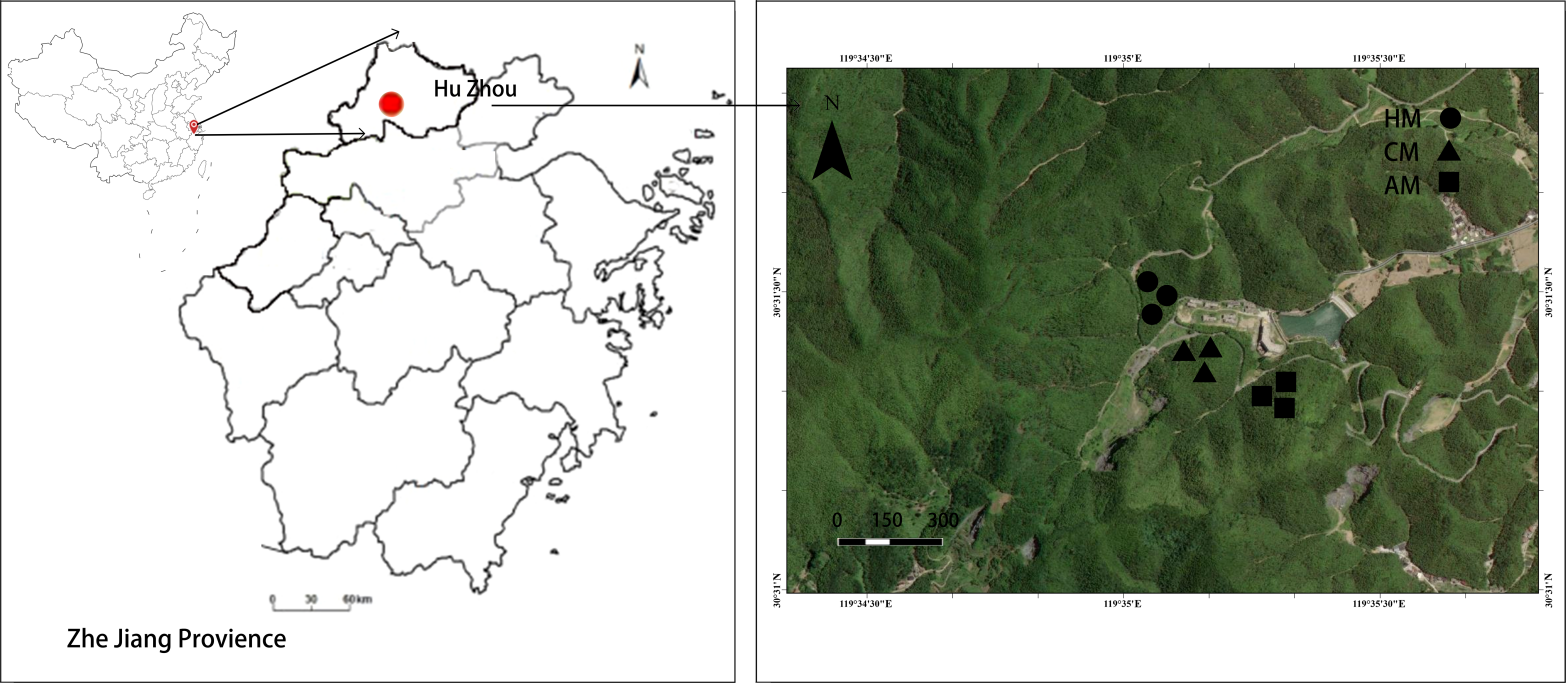
Location of the study area and sampling sites in the abandonment management (no fertilization and no-tillage treatment, AM), conventional management (biennial fertilization, CM), and high-intensity management (annual fertilization and planting with Chinese herbs under the Moso bamboo, HM) Moso bamboo plantations.

Within the homogeneous stand, three adjacent management regimes (AM, CM, and HM) were identified based on historical practice monitoring in Anji County. For each management type, three 20 m × 20 m representative plots were randomly established and separated by at least 10 m to minimize cross-effects. This design ensured that each management treatment was replicated across three distinct plantations, with three plots per plot, totaling nine experimental units (3 managements × 3 plots) (**Figure 2**).

### 2.2 Plant and soil sampling and processing

During the peak of the 2023 growing season, green leaf samples were gathered. To ensure representation, we randomly chose ten healthy and mature bamboo plants that matched the stand’s average diameter at breast height and stem height. These Moso bamboos were labelled, and a representative branch from each bamboo exposed to sunlight was collected from the upper two-thirds of the canopy using high pruning shears. We immediately separated the matured green leaves of the current year based on leaf properties such as colour, and morphology from these bamboo branches. Then, we pooled the intact green leaves within each subplot to form a mixed sample weighing approximately 500 g. Within each plot, five litter traps (1 m × 1 m) were placed to collect the freshly fallen senescent leaves of Moso bamboo. The freshly fallen senesced leaves were collected from the litter traps in October 2023. Any visibly damaged leaves caused by fungi, pests, diseases, or physical factors were excluded from this study.

Ten rhizome-system roots were selected in each plot. We used a pickaxe and spade to loosen the mineral soil around the rhizome root. Subsequently, we carefully excised intact fine roots, encompassing at least 1^st^ to 5^th^ orders, from the rhizome system of Moso bamboo. Each root sample was split into two parts. The first subsample was carefully rinsed with deionized water to eliminate adhering soil and promptly preserved in formalin-aceto-alcohol to analyze anatomical characteristics. The second subsample was placed in valve bags to measure the traits. Additionally, soil samples were collected concurrently with the root sampling, sieved through a 2 mm mesh, and sealed in valve bags. The root and soil samples were maintained at a constant temperature in a thermostat during their transportation to the laboratory.

### 2.3 Plant traits and soil property measurements

All root samples were washed using distilled water and dried before further analysis. Fine roots were graded using tools such as magnifying glasses, vernier callipers, and forceps. We dissected roots according to the branching-order classification method (Pregitzer et al., 2002), keeping only the finest 1^st^- and 2^nd^ order roots for our experiment as “absorptive roots” in following. This designation aligns with anatomical evidence showing >85% cortical parenchyma retention in these orders (Guo et al., 2008), and physiological studies demonstrating 78-95% of short-term (< 72 h) nutrient uptake occurs in 1^st^/2^nd^ orders (McCormack et al., 2015). Thus, we keep the two order roots to ensure methodological consistency with standardized protocols across root ecology studies (McCormack et al., 2015; Jiang et al., 2021; Zheng et al., 2024).

Absorptive roots were spread out evenly on glass plates to prevent overlapping and then scanned using a plant image analyzer (manufactured by Seiko Epson Corporation, located in Suwa, Nagano, Japan) at a resolution of 400 dpi. To eliminate any background noise from the root images, we employed Adobe PHOTOSHOP CC 2017 software (developed by Adobe Systems Inc., San Jose, CA, USA). Then, WinRHIZO Arabidopsis version 2012b (Regent Instruments Inc., Quebec City, Quebec, Canada) was used to measure the root diameter (RD) and root length (RL). Subsequently, the roots were dried in an oven at 60°C (48 h) to measure their biomass. Following this, specific root length (SRL), defined as the ratio of root length to root dry mass, and root tissue density (RTD), calculated as the ratio of root dry mass to root volume, were determined. We computed the branching intensity (BI) by dividing the total number of first and second-order roots by the length of the third-order roots (Kong et al., 2014; Jiang et al., 2023). We selected more than 20 segments from the formalin-aceto-alcohol solution for measuring anatomical traits. The segments were stained with safranin-fast green, dehydrated in a set of alcohol solutions, and embedded in paraffin. Then, root sections (8-μm-thick) were prepared, photographed using a compound microscope (DM2500, DFC450; Leica, Wetzlar, Germany) and measured for cortex thickness (CT) using IMAGEJ (NIH Image, Bethesda, MD, USA) (Zheng et al., 2024).

The dried root and leaf samples were powdered using a Retsch MM 400 mixer mill (manufactured by Retsch GmbH in Haan, Germany) for chemical analysis. The elemental analyzer Vario Macro cube (produced by Elementar Analysensysteme GmbH in Langenselbold, Germany) was used to determine the concentrations of carbon (C) and nitrogen (N). After microwave digestion with H_2_SO_4_, the phosphorus (P) concentration was measured using inductively coupled plasma mass spectrometry (Optima 5300 DV; manufactured by Perkin Elmer in Waltham, MA, USA). The concentration of non-structural carbon (NSC) was derived by summing the concentrations of soluble sugars and starch. Specifically, the concentration of soluble sugars was measured using the anthrone-colorimetric technique, and the starch concentration was assayed through the application of the dilute acid hydrolysis method.

Total soil C, N and P concentrations were determined using the same methods for analyzing plant nutrients. Soil ammonium and nitrate were extracted from 10 grams of soil using 50 milliliters of 2 mol L^−1^ KCl solution. Additionally, soil available phosphorus (AP) was extracted from 5 grams of soil using 50 milliliters of a KCl/NH_4_F solution containing 0.025 mol L^−1^ KCl and 0.03 mol L^−1^ NH_4_F. The concentrations of these nutrients were then determined using a continuous-flow auto-analyzer (model Autoanalyser 3, manufactured by Bran and Luebbe in Germany). Soil pH was measured at a soil: water ratio of 1: 2.5 (w/v) using a digital pH metre (Mettler Toledo, Greifensee, Switzerland). Soil microbial biomass carbon (MBC) and nitrogen (MBN) were quantified via the chloroform fumigation-extraction method (Vance et al., 1987; Perakis and Hedin, 2002). C and N concentrations in fumigated and non-fumigated soil extracts were analyzed using a Multi C/N 3000 Analyzer (Analytik Jena, Germany). Microbial biomass values were derived from differences in extractable C and N between treatments, adjusted by extraction coefficients of 0.45 (MBC) and 0.25 (MBN) (Malchair and Carnol, 2009).

### 2.4 Root and leaf nutrient acquisition

We estimated the potential for nutrient absorption in the roots by utilizing the root biomass and the root-soil accumulation factor (RSAF) (Kou et al., 2017; Jiang et al., 2023). The root biomass was estimated based on the key root traits (RD, SRL, RTD, BI, RL) that are closely associated with root construction and nutrient absorption (Hodge, 2004; Kong et al., 2014; Liese et al., 2017). The two indices were determined by thoroughly analyzing the scaling relationships among morphological characteristics of roots and the pathways of mycorrhizal symbiosis (Bergmann et al., 2020; Yan et al., 2022). For index I, we calculated the total length of absorptive roots by multiplying BI (biomass index) by RL (root length). Subsequently, we divided this total length by SRL (specific root length) to derive the biomass allocated per unit length of the third-order roots. For index II, our approach was grounded in the principles of cylindrical geometry (McCormack et al., 2015), we multiplied RTD by the volume of absorptive roots to obtain the biomass allocation per unit length of the third-order root. The RSAF was determined by dividing the nutrient concentration found in the absorptive roots by the concentration of inorganic nutrients present in the soil (Kou et al., 2017). More details are shown in Jiang et al. (2023).


$$\text{NAP-I }\left({\text{mg cm}}^{-1}\right)=\text{BI}\times \text{RL}/\text{SRL}\times {\text{N}}_{\text{AR}}/{\text{N}}_{\text{S}}\times 10$$

$$\text{NAP-II }\left({\text{mg cm}}^{-1}\right)=\text{BI}\times \text{RL}\times \text{RTD}\times \pi /4\times {\text{RD}}^{2}\times {\text{N}}_{\text{AR}}/{\text{N}}_{\text{S}}\times 10$$

$$\text{PAP-I }\left({\text{mg cm}}^{-1}\right)=\text{BI}\times \text{RL}/\text{SRL}\times {\text{P}}_{\text{AR}}/{\text{P}}_{\text{S}}\times 10$$

$$\text{PAP-II }\left({\text{mg cm}}^{-1}\right)=\text{BI}\times \text{RL}\times \text{RTD}\times \pi /4\times {\text{RD}}^{2}\times {\text{P}}_{\text{AR}}/{\text{P}}_{\text{S}}\times 10$$

BI represents the branching intensity, RL denotes the mean root length, and SRL stands for the specific root length. Additionally, RTD signifies root tissue density, while RD is the root diameter. N_AR_ and P_AR_ represent the concentrations of nitrogen and phosphorus, respectively, in the absorptive roots. On the other hand, Ns and Ps indicate the concentrations of inorganic nitrogen (comprising NH_4_^+^-N and NO_3_^−^-N) and available phosphorus, respectively, in the soil.

The nutrient resorption efficiency was calculated as the percentage decrease in nutrient concentrations from green leaves to senesced leaves (Aerts, 1997).


$$\text{NRE }\left(\%\right)=\left({\text{N}}_{\text{Green}}-{\text{N}}_{\text{Senesced}}\right)/{\text{N}}_{\text{Green}}\times 100$$

$$\text{PRE}\left(\%\right)=\left({\text{P}}_{\text{Green}}-{\text{P}}_{\text{Senesced}}\right)/{\text{P}}_{\text{Green}}\times 100$$

where NRE and PRE are the N and P resorption efficiencies, respectively. N_Green_, P_Green_, N_Senesced_, and P_Senesced_ are the N and P concentrations in green and senesced leaves, respectively.

Nitrogen resorption proficiency (NRP) and phosphorus resorption proficiency (PRP) are the resorption proficiencies of N and P, meaning the inverse of litter N and P concentrations (mg g^−1^), respectively. Lower litter nutrient concentrations correspond to higher nutrient resorption proficiencies and vice versa (Killingbeck, 1996).

Nitrogen resorption proficiency (NRP) and phosphorus resorption proficiency (PRP) refer to the ability of plants to resorb nitrogen (N) and phosphorus (P), respectively. These proficiencies are inversely proportional to the concentrations of N and P in litter (mg g^−1^). Hence, lower nutrient concentrations in litter indicate higher resorption proficiencies, and higher concentrations imply lower proficiencies (Killingbeck, 1996).

### 2.5 Statistical analyses

All variables were assessed for normality using the Shapiro-Wilk test (α = 0.05) and log-transformed when necessary for all the measured values. We assessed the correlation between root nutrient uptake and leaf nutrient reclamation using Pearson’s correlation coefficients. To establish a surrogate measure for root resource allocation, we conducted principal component analysis (PCA) on a set of root economic traits, encompassing RD, SRL, RTD, CT, and BI. Utilizing the first and second principal components derived from the PCA, we calculated the PC1 and PC2 scores for each site by employing the ‘PSYCH’ package within the R programming environment. We then determined the bivariate relationships between major axis scores and nutrient absorption potential and nutrient resorption for all the sites using the package ‘SMATR’ package in R.

Considering Moso bamboo species characteristics, it typically needs conventional management to maintain bamboo productivity, thus we considered CM treatment as a control treatment. Based on this experience, AM and HM, as the different treatments, correspond to natural succession and high-intensity manual treatment, respectively.

An analysis of variance (ANOVA) was used to test the effects of different managements on root traits, nutrient absorption potential as well as nutrient resorption. Means were compared for statistical significance using Tukey’s HSD (honestly significant difference) test. Statistical significance was determined at a *P*-value of less than 0.05. All statistical analyses were conducted using R software (v.4.1.3; https://www.R-project.org/). All figures were created using Origin 2021 software.

## 3 Results

### 3.1 Root nutrient absorption and leaf nutrient resorption

Across the three treatments, the values of root nutrient absorption potentials were similar when calculating via the two indices (NAP-I = 0.20 ± 0.10 mg cm^-1^, NAP-II = 0.17 ± 0.08 mg cm^-1^; PAP-I = 0.13 ± 0.10 mg cm^-1^, PAP-II = 0.10 ± 0.07 mg cm^-1^; **Table 1**). The average N and P resorption efficiencies were 48% (CV = 22%) and 62% (CV = 15%), respectively (**Table 1**).

**Table 1:** Summary of the root and leaf traits and processes of Moso bamboo across the three different managements (*n* = 9).

Trait or processes (abbreviation, unit)	Mean	SD	Min	Max	CV (%)
Root diameter (SD, mm)	0.29	0.01	0.26	0.36	3.45
Specific root length (SRL, m g^-1^)	13.63	1.20	8.27	19.09	8.80
Root tissue density (RTD, g cm^-3^)	0.98	0.04	0.79	1.12	3.93
Branching intensity (BI, tips cm^-1^)	3.20	0.07	2.98	3.54	2.18
Stele diameter (SD, mm)	0.17	0.01	0.11	0.21	5.44
Cortex thickness (CT, mm)	0.21	0.01	0.18	0.23	2.71
Mycorrhizal colonization rate (MCR, %)	46.12	4.72	26.67	61.11	10.24
Root nitrogen content (N_Root_, mg g^-1^)	15.39	0.81	12.50	19.70	5.24
Root phosphorus content (P_Root_, mg g^-1^)	1.94	0.17	1.38	2.72	8.67
Root non-structural carbon (NSC_Root_, mg g^-1^)	8.61	0.40	6.41	10.77	4.66
Green leaf Nitrogen (N_Green_, mg g^-1^)	60.23	3.94	46.10	75.50	6.54
Green leaf phosphorus (P_Green_, mg g^-1^)	5.73	0.45	3.88	7.70	7.87
Green leaf non-structural carbon (NSC_Green_, mg g^-1^)	21.78	0.94	18.04	25.49	4.33
Senesced leaf nitrogen (N_Senesced_, mg g^-1^)	29.83	1.86	21.80	39.10	6.23
Senesced leaf phosphorus (P_Senesced_, mg g^-1^)	2.09	0.09	1.78	2.48	4.37
Senesced leaf non-structural carbon (NSC_Senesced_, mg g^-1^)	9.83	0.87	7.03	14.14	8.85
Nitrogen absorption potential (NAP-I, mg cm^-1^)	0.18	0.03	0.08	0.31	17.92
Nitrogen absorption potential (NAP-II, mg cm^-1^)	0.16	0.03	0.06	0.27	17.89
Phosphorus absorption potential (PAP-I, mg cm^-1^)	0.11	0.03	0.05	0.29	25.03
Nitrogen absorption potential (PAP-II, mg cm^-1^)	0.09	0.02	0.05	0.22	22.40
Nitrogen resorption efficiency (NRE, %)	47.87	10.45	29.28	60.65	21.82
Phosphorus resorption efficiency (PRE, %)	61.86	9.29	43.30	71.69	15.02

**Figure 3:**
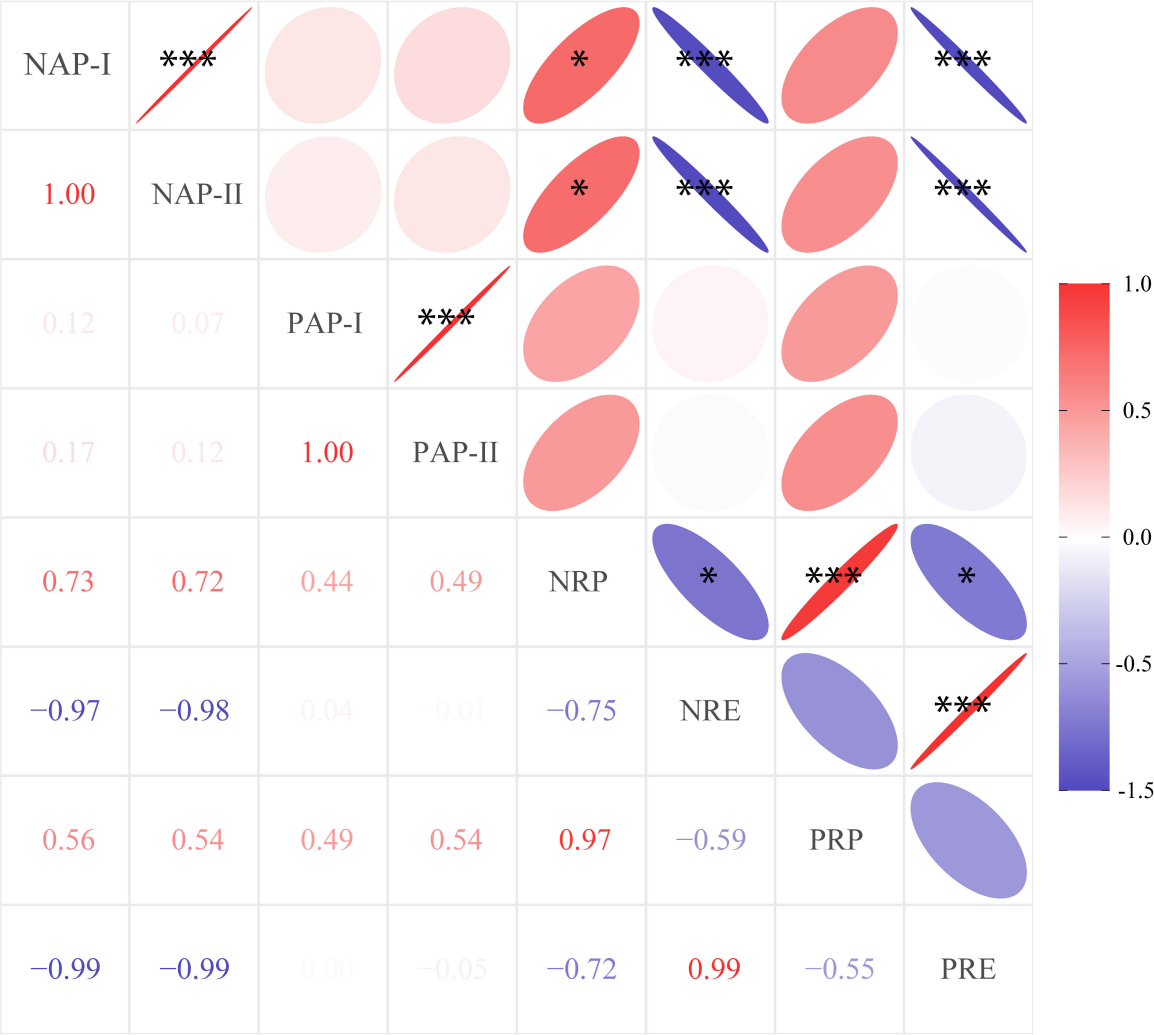
Pairwise correlations between nutrient-associated processes for Moso bamboo specie, regardless of management (*n* = 9). The number in the square is the correlation coefficient. Asterisks indicate a significant relationship (*P< 0.05, **P< 0.01, ***P< 0.001). NAP-I and NAP-II are the nitrogen absorption potential, PAP-I and PAP-II are the phosphorus absorption potential, NRP is the nitrogen resorption proficiency (the negative of nitrogen concentration in leaf litter was used here), NRE is the nitrogen resorption efficiency, PRP is the phosphorus resorption proficiency (the negative of phosphorus concentration in leaf litter was used here), and PRE is the phosphorus resorption efficiency.

We used linear analysis to explore the bivariate relationships between root nutrient absorption and leaf nutrient resorption and found that both NAP-I and NAP-II were negatively correlated with nitrogen resorption efficiency (NRE) (*R^2^* = 0.75, *P* = 0.003 for NAP-I; *R^2^* = 0.80, *P* = 0.001 for NAP-II; **Figure 3**) rather than nitrogen resorption proficiency (NRP) (*P* > 0.05). For P element, P absorption potential (PAP-I and PAP-II) were no significantly correlated with P resorption (PRE or PRP) (**Figure 3**).

### 3.2 Traits and their relationships with root nutrient absorption and leaf nutrient resorption

The traits of Moso bamboo varied considerably across the three different management practices (**Table 1**). The largest variation in the morphological, anatomy, and chemical traits of absorptive roots were obtained for specific root length (SRL) (coefficient of variation (CV) = 26%), mycorrhizal colonization rate (MCR) (CV = 31%), and non-structural carbon (NSC_Root_) (CV = 27%), respectively. For green and senesced leaves, litter N and P concentrations showed an approximately two- and three-fold variation across the three managements, respectively (**Table 1**).

**Figure 4:**
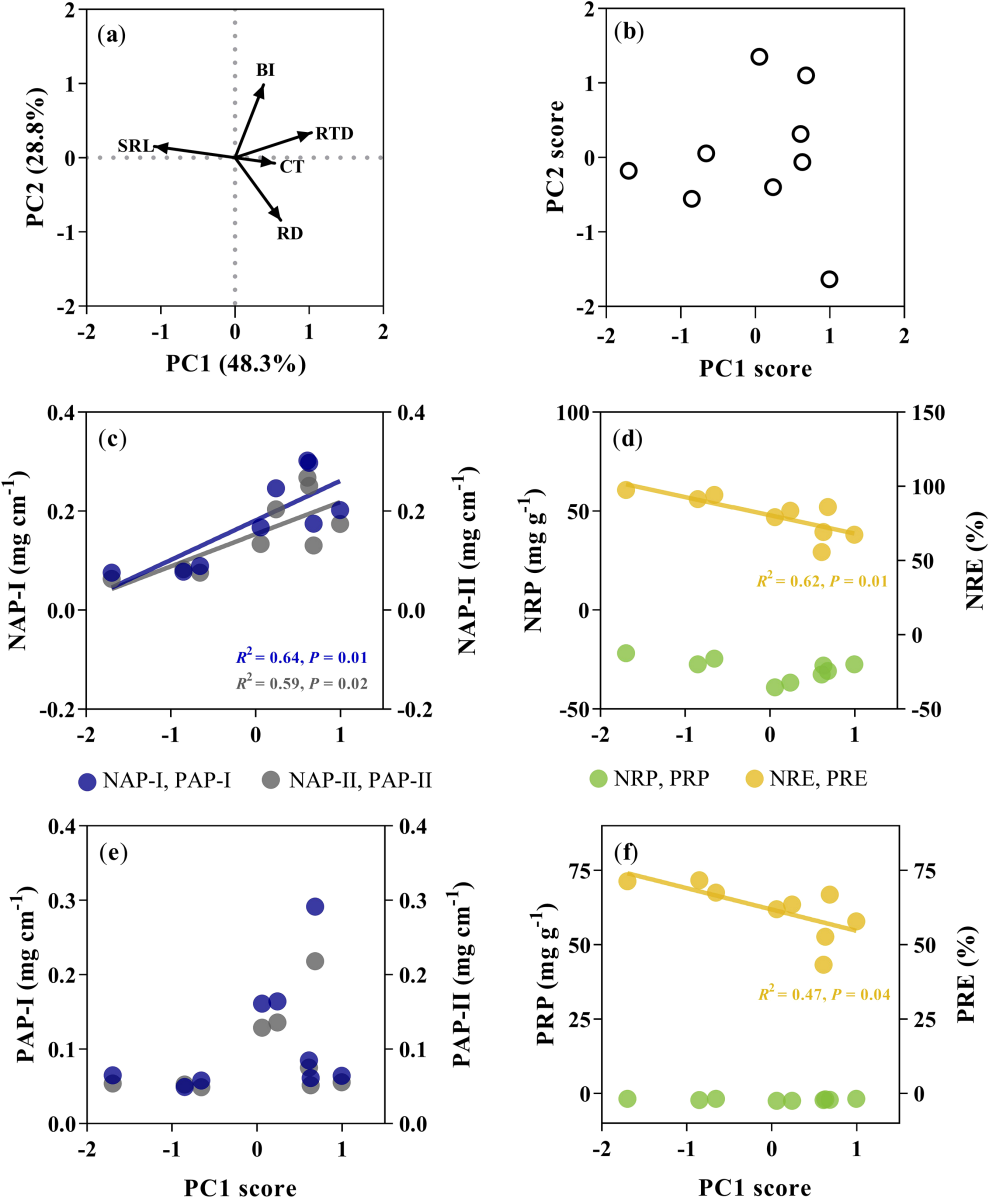
Principal component analysis (PCA) for root traits and relationships of the loading score of the first principal component analysis (PC1) with the nutrient-associated processes. The PCA conducted five key root traits, including root diameter (RD), specific root length (SRL), root tissue density (RTD), branching intensity (BI), and cortex thickness (CT) for Moso bamboo specie. **(a)** Trait loading biplot. **(b)** individual specie distribution in the one-dimensional trait space. Relationships of PC1 score with the nitrogen absorption potential (**c**, NAP-I (blue dots), NAP-II (gray dots), nitrogen resorption proficiency (the negative of nitrogen concentration in leaf litter was used here) and nitrogen resorption efficiency (**d**, NRP (green dots), NRE (yellow dots)), phosphorus absorption potential (**e**, PAP-I (blue dots); b, PAP-II (gray dots)), phosphorus resorption proficiency (the negative of nitrogen concentration in leaf litter was used here) and phosphorus resorption efficiency (**f**, PRP (green dots), PRE (yellow dots).

For all managements, the PCA conducted on the five root traits accounted for 77% of the variation in the first two axes (**Figure 4a**). The PC1 was correlated with SRL and root tissue density (RTD), and PC2 was correlated with root diameter (RD) and branching intensity (BI). The PC1 score rather than the PC2 score was significantly positively correlated with N absorption potential (*R^2^* = 0.64, *P* = 0.010 for NAP-I; *R^2^* = 0.59, *P* = 0.016 for NAP-II; **Figure 4c**), and negatively correlated with NRE (*R^2^* = 0.62, *P* = 0.012; **Figure 4d**; **Supplementary Figure S1**). The PC1 score was only negatively correlated with PRE (*R^2^* = 0.47, *P* = 0.04; **Figure 4f**).

### 3.3 Effects of different managements on traits and processes

**Figure 5:**
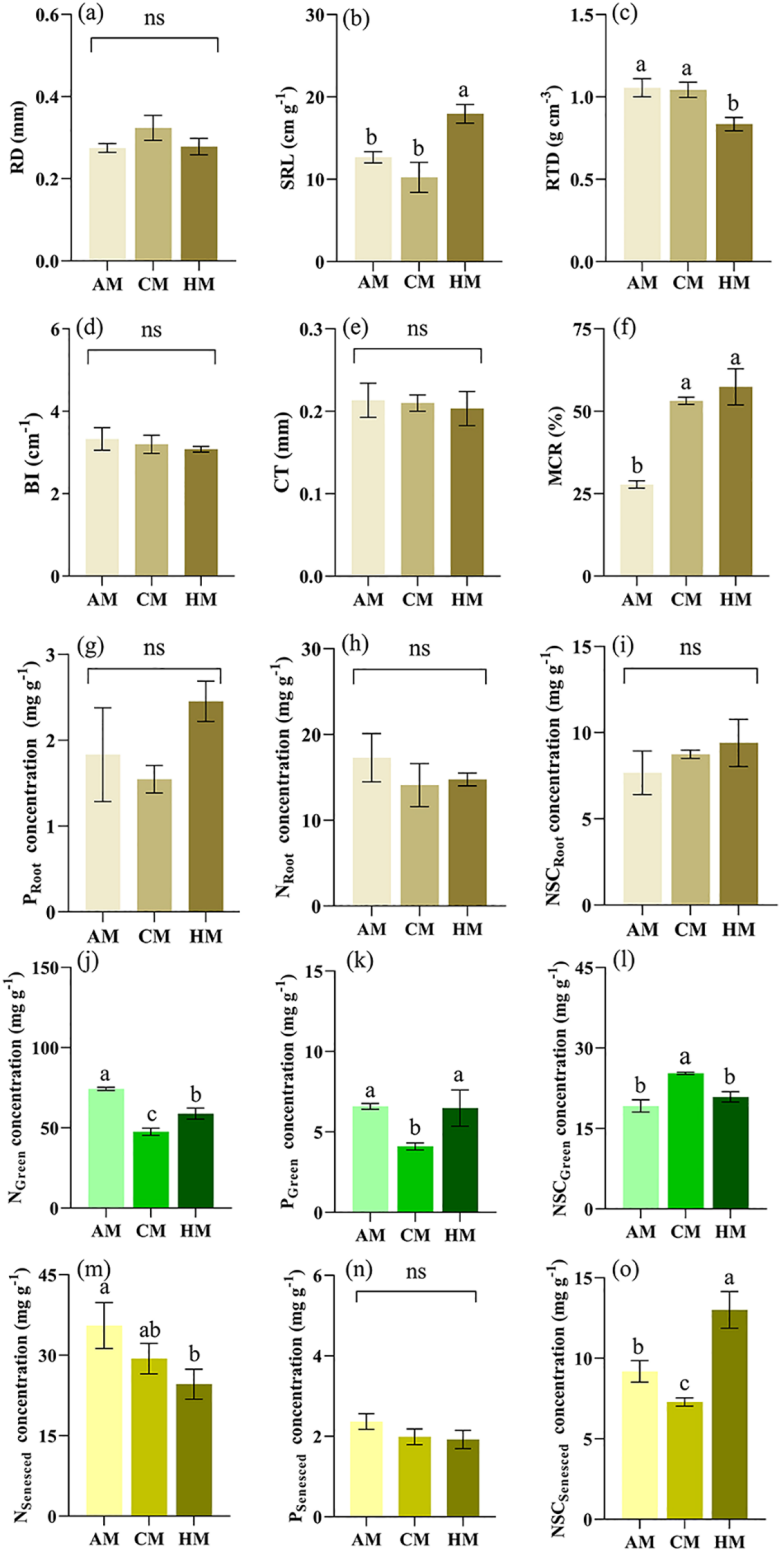
Influence of different managements on root and leaf traits. Values are mean ± SE (*n* = 3). Significant differences between means were determined using Tukey's honestly significant difference test. Different letters indicate significant differences among treatments (*P* < 0.05). AM, abandonment management; CM, conventional management; HM, high-intensity management; **(a) ** RD, root diameter; **(b)** SRL, specific root length; **(c)** RTD, root tissue density; **(d)** BI, branching intensity; **(e) **CT, cortex thickness; **(f)** MCR, mycorrhizal colonization rate; **(g)** P_Root_, phosphorus in root; **(h)** N_Root_, nitrogen in root; **(i)** NSC_Root_, non-structural carbon in root; **(j)** N_Green_, nitrogen in green leaf; **(k)** P_Green_, phosphorus in green leaf; **(l)** NSC_Green_, non-structural carbon in green leaf; **(m)** N_Senesced_, nitrogen in senesced leaf; **(n)** P_Senesced_, phosphorus in senesced leaf; **(o)** NSC_Senesced_, non-structural carbon in senesced leaf.

For morphological traits of the absorptive root, AM and CM treatments significantly decreased SRL (*P* = 0.001; **Figure 5b**), but increased RTD (*P* = 0.002; **Figure 5c**), respectively, compared to HM treatment. For anatomy traits of absorptive root, cortex thickness (CT) showed no significant differences among the three treatments (*P* > 0.05; **Figure 5e**). However, MCR was lowest in AM treatment, compared with CM and HM treatments (*P*< 0.001; **Figure 5f**). For chemical traits of absorptive roots (N_Root_, P_Root_, NSC_Root_), there were no significant differences among the three treatments (**Figures 5g–i**).

Green leaves had significantly lower nitrogen (N_Green_) and phosphorus (P_Green_) concentrations in CM treatment than that in HM and AM treatments (*P*< 0.001 for N_Green_; *P* = 0.006 for P_Green_; **Figures 5j, k**), whereas the opposite trend was observed for non-structural carbon in green leaves (NSC_Green_) (*P*< 0.001 for NSC_Green_; **Figure 5l**). Senesced leaves showed higher concentration of N in AM than in CM and HM treatments (*P* = 0.021; **Figure 5m**), while the concentration of non-structural carbon (NSC_Senesced_) in HM significantly higher in AM and CM treatments (*P*< 0.001; **Figure 5o**).

**Figure 6:**
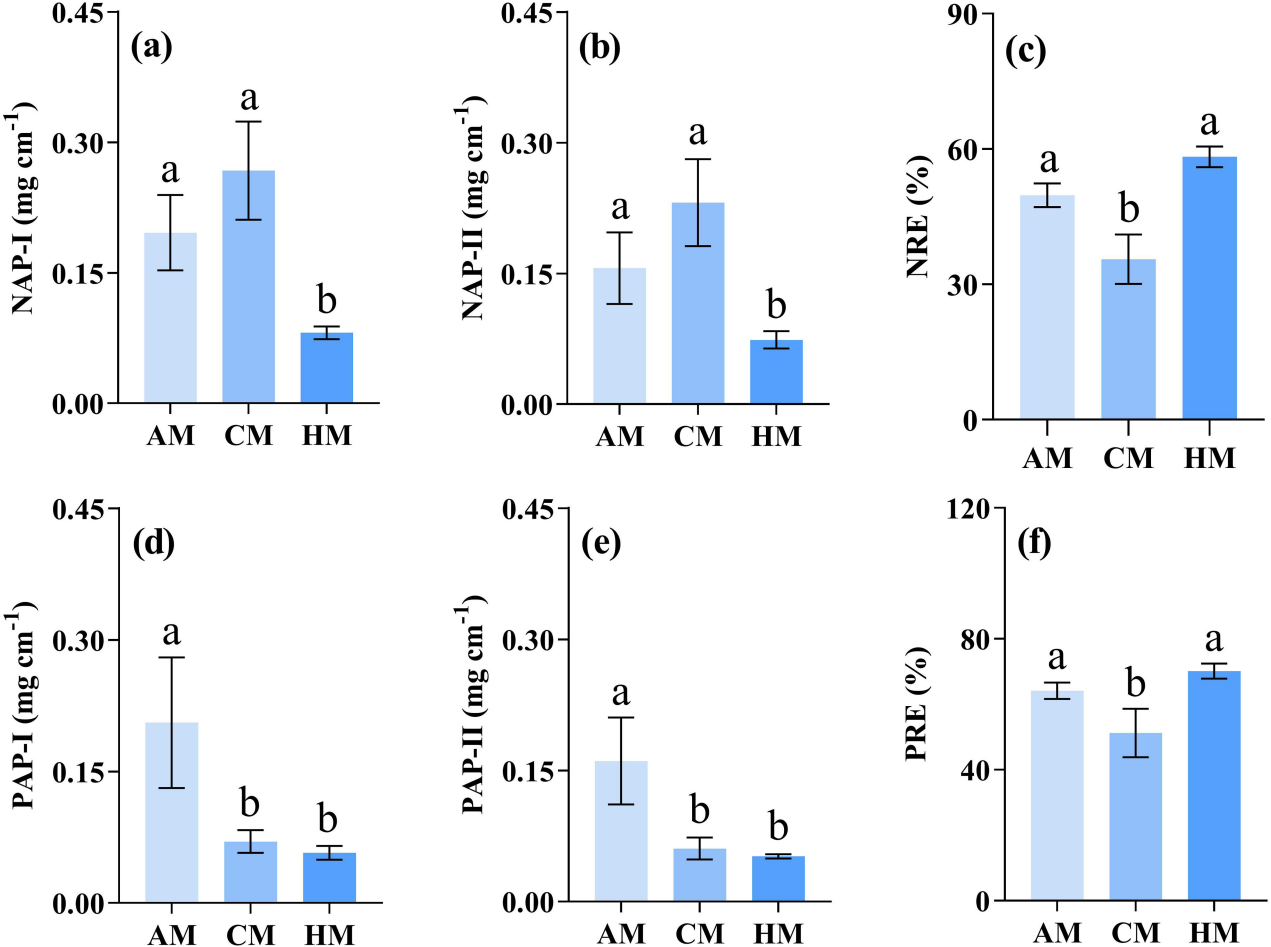
Influence of different managements on nutrient-associated processes. Values are meant ± SE (*n* = 3). Significant differences between means were determined using Tukey's honestly significant difference test. Different letters indicate significant differences among treatments (*P* < 0.05). AM, abandonment management; CM, conventional management; HM, high-intensity management; **(a)** NAP-I and **(b)** NAP-II, nitrogen absorption potential; **(c)** NRE, nitrogen resorption efficiency; **(d) **PAP-I and **(e) **PAP-II, phosphorus absorption potential; **(f)** PRE, phosphorus resorption efficiency.

The HM treatment significantly decreased the values of nutrient absorption potentials compared to CM or AM treatments (*P* = 0.004 for NAP-I, *P* = 0.007 for NAP-II, *P* = 0.011 for PAP-I, *P* = 0.007 for PAP-II; **Figures 6a, b, d, e**). Nutrient resorption efficiencies (NRE and PRE) were significantly lower in CM treatment than in HM and AM treatments (*P* = 0.017 for NRE, *P* = 0.007 for PRE; **Figures 6c, f**).

## 4 Discussion

### 4.1 A trade-off between N absorption via rhizome-system root and N resorption by leaves exists

We previously reported that the root nutrient absorption (get) - leaf nutrient resorption (save) - leaf litter decomposition (return) ‘GSR’ continuum exists and runs on P economy based on 15 co-occurring woody tree species in subtropical forests (Jiang et al., 2023). However, for clonal species, the ubiquity of this continuum remains uncertain. Here, we selected a woody rhizomatous plant, Moso bamboo, which differs markedly from woody tree species in that it reproduces asexually during the explosive growth period and produces new individuals that are genetically identical to the parent, forming clonal communities (Song et al., 2017; Li L. et al., 2023). These clonal individuals are often linked together, allowing them to share resources, suggesting that plant-centred processes are closely related. Thus, we speculate that there may be an active trade-off between nutrient acquisition by the roots and leaf nutrient resorption.

In line with our first hypothesis, Moso bamboo does indeed exhibit a trade-off between nutrient absorption and nutrient resorption, reflecting a cost-benefit accounting likely other woody tree species (Brant and Chen, 2015; Li Q. et al., 2023; Jiang et al., 2023). Energy costs are generally considered to be an indicator that plants tend to choose between two nutrient acquisition pathways: root uptake from the soil and nutrient resorption from senescent leaves (Wright and Westoby, 2003; Wang et al., 2014). For example, tall perennial herbs rather than short herbaceous conducted positive relationships between root acquires and leaf resorption, but mowing eliminated this relationship (Li Q. et al., 2023). However, woody species preferentially use nutrient resorption strategy under nutrient-deficient conditions (Hodge, 2004), but switch to use root uptake strategy with more available nutrients in soils (Kou et al., 2017). The unique clonal nature of Moso bamboo further confirmed the fundamental active balance. Such absorption-resorption trades-offs may represent a broader ecological mechanism. Our findings provide a novel perspective on the resource allocation strategies in clonal species and emphasize the critical role of both belowground and aboveground processes in shaping their nutrient cycling.

Interestingly, this active trade-off primarily focused on the N rather than P economy (**Figure 3**), suggesting that N plays a pivotal role in driving nutrient acquisition of Moso bamboo. This may be related to the explosive growth of Moso bamboo, particularly during the spring shoot emergence and leaf expansion stages (Chen et al., 2022). During these stages, the increasing demand for N may support the bamboo’s photosynthesis and overall biomass accumulation, as well as being a major component of proteins, chlorophyll, and various metabolic enzymes (Lebauer and Treseder, 2008; Yang et al., 2022). In addition, the high N demand may be compensated via root uptake strategy, as the larger and more complex rhizome systems play a critical role in nutrient absorption, especially under the background of decreasing N available (Chaparro et al., 2014; Mason et al., 2022). It’s worth noting that the two values of N absorption potential were negatively correlated with NRE rather than NRP. The NRE is calculated as the relative percentage reduction in N concentration from green to the senesced leaves (Killingbeck, 1996). This is an efficient response to N retention in living leaves, suggesting a close link between NRE and N uptake via the root system in plant-centred processes, especially for cloned species. However, the NRP represents the absolute N concentration in the senesced leaves (Killingbeck, 1996), which may be correlated with legacy effects such as litter decomposition (Jiang et al., 2023).

Unlike N, which is taken up by roots in both inorganic and organic forms, the P absorbed by roots is usually inorganic and requires considerable energy investment (Schachtman et al., 1998; Lambers, 2022). Based on the cost-benefit economics, plants may choose multiple pathways to acquire P to meet P demand, especially in P-limited subtropical forests (Lambers, 2022). and the positive correlations between P absorption potential and P resorption prove the multi-approach strategy. These results suggest that root P absorption and leaf P resorption may be comparable important in the P requirements of Moso bamboo, but more experiments and data are needed to confirm this. In addition, abundant research has demonstrated the key role of arbuscular mycorrhizal fungi or phosphate-solubilizing microorganisms in transforming organic P into inorganic P (Lambers, 2022; Zhu et al., 2024), thus expanding the scope and strength of root uptake of P. Alternatively, P in leaves can be acquired by resorption. Previous studies have also verified that more P is allocated to leaves than to roots (Wang et al., 2022). Taken together, these results suggest that P acquisition by roots and leaves may be a synergistic cooperation rather than a trade-off, but more evidence is needed in future studies.

### 4.2 Root traits linked with nutrient-associated processes

Root trait syndromes can be regarded as the most efficient tools for predicting root processes such as nutrient acquisition, chemical defense, and root decomposition (Yaffar et al., 2022; Yan et al., 2022; Zheng et al., 2024). Thus, we conducted the key root traits of bamboo based on the PCA, and these traits can be reflected by a bi-dimensional root economic space, in line with previous findings for woody tree species (Yan et al., 2022; Zheng et al., 2024). The first principal component (PC1) axis is symbolized by SRL and RTD, representing a ‘lifespan gradient’ showing the investment in the bamboo growth. Roots with higher SRL and lower RTD are typically associated with rapid nutrient uptake in nutrient-rich environments, while the opposite combination supports persistence in nutrient-poor conditions (Comas et al., 2013). The second principal component (PC2) axis indicated by BI and CT, representing a ‘symbiosis gradient’ showing the investment in their fungal partners. Larger CT and smaller BI indicate a fungal-symbiosis dependency in root strategy, where plants invest more C in expanding cortical space rather than in expanding more ramified roots to increase habitat availability for arbuscular mycorrhizal fungi, which are better able to exploit soil volume through fungal symbiosis (Freschet and Roumet, 2017; Wen et al., 2019; Yan et al., 2022). Therefore, identification of bamboo rhizome root trait syndromes is essential for understanding bamboo forest ecosystem processes and predicting bamboo responses to the effects of different management regimes.

Moreover, we addressed the correlations between root trait syndromes and processes. One of the results showed that SRL and RTD of bamboo roots can jointly predict root N absorption and N resorption, paving the way for targeted ecosystem management. For example, a higher intensity management decreased the SRL, and specific root area, but increased the RTD (Ni and Su, 2024). However, the different strip clearcutting had no significant effects on root traits (Zheng et al., 2021). N fertilization has been observed to stimulate root growth and increase root length (Shi et al., 2024). Thus, by modifying traits such as SRL or RTD through selective breeding or management interventions, it may be possible to optimize root contributions to N cycling and, ultimately increase the productivity of bamboo ecosystems. These insights provided a theory basis for sustainable land management practices of Moso bamboo.

Besides, the relationships between root traits and processes may be reflected by physiological mechanism. For example, under sufficient nutrient conditions, Moso bamboo preferentially invests C in constructing absorptive roots (high root biomass and root length density) to exploit soil available nutrient (Ni and Su, 2024), reducing reliance on leaf resorption—a strategy aligned with the ‘acquisitive’ end of the root economics spectrum (Reich, 2014). Conversely, under nutrient-limited conditions, C is redirected to enhance resorption enzymes or related gene expression, maximizing internal nutrient recycling (Paul and Pellny, 2003). Although our data lack direct evidence, future studies should quantify the relative contributions of root uptake versus clonal redistribution to nutrient allocation dynamics, such as ^15^N tracing and molecular genetics.

### 4.3 Effects of different management on the root traits and the linked processes

In support of our third hypothesis, different managements partially influenced bamboo root traits and associated processes. The increased SRL and reduced RTD under HM suggest a shift toward root acquisitive strategies, likely driven by intensified belowground competition with understory herbs. This aligns with the ‘lifespan gradient’ framework, where higher SRL enhances nutrient foraging capacity in resource-heterogeneous environments (Yan et al., 2022). These findings for bamboo species were not consistent with general rules for woody tree species, where higher SRL and lower RTD are typically associated with rapid nutrient uptake in nutrient-rich environments (Comas et al., 2013). These contradictory results may be due to the changing rhizome system in fertile soils. In our study, the HM site was planted with Chinese herbs and fertilized with compound fertilizer, which improves soil fertility but may also increase competition. Thus, the Moso bamboo rhizome system under HM treatment may exhibit increasing SRL to extend root absorption range to escape the competition and intensify nutrient resorption by leaves to meet higher production. Interestingly, the N and P concentrations significantly increased in green leaves but decreased in senesced leaves under HM treatment, respectively, whereas the NSC concentration was inversely affected. These contrasting patterns further highlight the intricate relationship between nutrient allocation and NSC in bamboo (Song X. et al., 2016). The increased N and P concentrations in green leaves suggest enhanced nutrient uptake and utilization, possibly due to the increased SRL to acquire nutrients in a competitive environment under HM treatment, which is not consistent with the lower SRL found in Ni and Su (2024). However, the reduced N and P concentrations in senesced leaves, coupled with the higher NSC concentrations, suggest that bamboo under HM treatment may prioritize C storage in leaves for future growth, while simultaneously enhancing nutrient resorption during senescence to conserve essential nutrients for the upcoming growth cycle. These results suggest that, under certain management practices, bamboo exhibits adaptive strategies to balance nutrient acquisition and C storage, thereby improving its growth and resilience in nutrient-competitive environments.

On the other hand, AM treatment had slight effects on key root traits compared to CM treatment, except for a significant decrease in mycorrhizal colonization rate. The absence of active management practices may explain the lower MCR, as it increases the abundance of microbe with higher microbial biomass carbon (**Supplementary Figure S2**) and reduces the symbiotic associations. Although the AM treatment had a minimal effect on root morphological traits compared to the CM treatment, it notably altered the N and P concentrations in the leaves. Likely to HM treatment, AM treatment significantly increased the N and P concentrations in green leaves while decreasing the NSC levels. In terms of nutrient resorption, AM treatment, like HM treatment, enhanced the nitrogen and phosphorus resorption efficiency, suggesting that abandonment practices may rely more on leaf nutrient resorption to maintain resource input, compensating for the lack of active root system management. For example, studies on abandoned forests have shown that reduced disturbance often leads to greater nutrient retention within leaf tissues, improving nutrient-use efficiency to compensate for lower nutrient input from external sources (Deng et al., 2020). Similarly, plant species often observe a decrease in nutrient resorption with increasing fertilization conditions (Yuan and Chen, 2015). These findings suggest that bamboo under AM treatment likely adopts similar strategies, leveraging efficient nutrient recycling via leaves to ensure growth despite reduced management intervention. Overall, these management practices highlight the potential of root traits as a key lever for optimizing ecosystem services such as nutrient cycling and C sequestration in bamboo plantations, thereby supporting sustainable land use and forest management practices.

Moso bamboo, as a clonal species, relies heavily on its rhizome system for resource storage and translocation between ramets. The rapid spring shoot growth, characterized by explosive biomass accumulation, likely depends on both immediate soil N uptake (via root-soil interfacial absorption of NH_4_^+^/NO_3_^−^) and remobilization of stored N from rhizomes, which may originate from historical root absorption or leaf nutrient resorption (Song Q. et al., 2016; Chen et al., 2022). Interestingly, soil NH_4_^+^ and SRL jointly explained root-leaf N allocation in Moso bamboo based on the redundancy analysis, especially under HM treatments (**Supplementary Table S1**; **Supplementary Figure S3**), suggesting that fertilization may alter soil N availability and plant physiological adjustments to adapt environment changes. Moreover, the positive relationships between MBC and litter N concentration (**Supplementary Table S2**), in line with theory that litter qualities influence microbial community (Berg and McClaugherty, 2014; Luan J. et al.,2024). Under HM treatment, the litter N concentration and MBC both significantly decreased, and N resorption increased, which together indirectly explained that Moso bamboo may tend to get nutrient via multiple strategies such as leaf nutrient resorption and root nutrient absorption to subsidize shoot growth under potential competition conditions. However, our experimental design did not directly measure microbial community and explicitly quantify the proportional contribution of clonal N redistribution versus direct root uptake to shoot N demands—a limitation we acknowledge. Future studies will employ ^15^N isotopic tracing to partition shoot N sources, correlate rhizome N reserves with shoot growth rates, and analyze N-metabolism gene expression patterns to unravel regulatory mechanisms. These approaches will clarify the interplay between clonal integration and localized nutrient strategies in Moso bamboo.

### 4.4 Implications and future research

This study provides a robust foundation for balancing resource acquisition between the above-ground and below-ground parts of the Moso bamboo, especially due to its clonal nature. The results show that Moso bamboo has an active trade-off between root nutrient uptake and leaf nutrient resorption, which extends the understanding of below- and above-ground resource allocation in plants and suggests that this mechanism may be more universal across diverse species. Furthermore, we confirmed the linkages between functional traits and processes, especially for root traits that reflect the lifespan dimension of the Moso bamboo root system, i.e. SRL and RTD. However, we focused on one zone, and the nutrient allocation patterns and gene expression between mother bamboo and shoot are unclear. Future studies should consider more scales and employ tissue-specific qPCR combined with ¹^5^N isotope tracing to spatially resolve gene activity and quantify their contributions to nitrogen redistribution efficiency, refining precision nutrient management strategies in bamboo forestry.

## Data Availability

The original contributions presented in the study are included in the article/**Supplementary Material**. Further inquiries can be directed to the corresponding author/s.

## References

[B1] Aerts R. (1997). Climate, leaf litter chemistry and leaf litter decomposition in terrestrial ecosystems: a triangular relationship. Oikos 79, 439–449. doi: 10.2307/3546886

[B2] Bardgett R. D. Mommer L. De Vries F. T. (2014). Going underground: Root traits as drivers of ecosystem processes. Trends Ecol. Evol. 29, 692–699. doi: 10.1016/j.tree.2014.10.006 25459399

[B3] Berg B. McClaugherty C. A. (2014). Plant Litter. Decomposition, Humus Formation, Carbon Sequestration. Berlin: Springer-Verlag. doi: 10.1007/978-3-662-05349-2

[B4] Bergmann J. Weigelt A. van der Plas F. Laughlin D. C. Kuyper T. M. Guerrero-Ramirez N.. (2020). The fungal collaboration gradient dominates the root economics space in plants. Sci. Adv. 6, eaba3756. doi: 10.1126/sciadv.aba3756 32937432 PMC7458448

[B5] Brant A. N. Chen H. Y. (2015). Patterns and mechanisms of nutrient resorption in plants. Crit. Rev. Plant Sci. 34, 471–486. doi: 10.1080/07352689.2015.1078611

[B6] Cai C. Fan S. Liu G. Wang S. M. Feng Y. (2018). Research and development advance of compound management of bamboo forests. World Bamboo. Rattan. 16, 50–55. doi: 10.13640/j.cnki.wbr.2018.05.011

[B7] Chaparro J. M. Badri D. V. Vivanco J. M. (2014). Rhizosphere microbiome assemblage is affected by plant development. ISME. J. 8, 790–803. doi: 10.1038/ismej.2013.196 24196324 PMC3960538

[B8] Chen M. Guo L. Ramakrishnan M. Fei Z. Vinod K. K. Ding Y.. (2022). Rapid growth of Moso bamboo (Phyllostachys edulis): Cellular roadmaps, transcriptome dynamics, and environmental factors. Plant Cell 34, 3577–3610. doi: 10.1093/plcell/koac193 35766883 PMC9516176

[B9] Chen W. Zeng H. Eissenstat D. M. Guo D. (2013). Variation of first-order root traits across climatic gradients and evolutionary trends in geological time. Global Ecol. Biogeogr. 22, 846–856. doi: 10.1111/geb.12048

[B10] Cleveland C. C. Houlton B. Z. Smith W. K. Marklein A. R. Reed S. C. Parton W.. (2013). Patterns of new versus recycled primary production in the terrestrial biosphere. Proc. Natl. Acad. Sci. 110, 12733–12737. doi: 10.1073/pnas.1302768110 23861492 PMC3732943

[B11] Comas L. H. Becker S. R. Cruz V. M. V. Byrne P. F. Dierig D. A. (2013). Root traits contributing to plant productivity under drought. Front. Plant Sci. 4. doi: 10.3389/fpls.2013.00442 PMC3817922 24204374

[B12] Deng X. Yin J. Xu L. Shi Y. Zhou G. Li Y.. (2020). Effects of abandonment management on soil C and N pools in Moso bamboo forests. Sci. Total. Environ. 729, 138949. doi: 10.1016/j.scitotenv.2020.138949 32387772

[B13] Fan P. Guo D. (2010). Slow decomposition of lower order roots: a key mechanism of root carbon and nutrient retention in the soil. Oecologia 163, 509–515. doi: 10.1007/s00442-009-1541-4 20058026

[B14] Freschet G. T. Roumet C. (2017). Sampling roots to capture plant and soil functions. Funct. Ecol. 31, 1506–1518. doi: 10.1111/nph.17072

[B15] Gu J. Xu Y. Dong X. Wang H. Wang Z. (2014). Root diameter variations explained by anatomy and phylogeny of 50 tropical and temperate tree species. Tree Physiol. 34, 415–425. doi: 10.1093/treephys/tpu019 24695727

[B16] Guo D. Xia M. Wei X. Chang W. Liu Y. Wang Z.. (2008). Anatomical traits associated with absorption and mycorrhizal colonization are linked to root branch order in twenty-three Chinese temperate tree species. New Phyto. 180, 673–683. doi: 10.1111/j.1469-8137.2008.02573.x 18657210

[B17] Hodge A. (2004). The plastic plant: Root responses to heterogeneous supplies of nutrients. New Phytol. 162, 9–24. doi: 10.1111/j.1469-8137.2004.01015.x

[B18] Huang K. Li Y. Hu J. Tang C. Zhang S. Fu S.. (2021). Rates of soil respiration components in response to inorganic and organic fertilizers in an intensively-managed Moso bamboo forest. Geoderma 403, 115212. doi: 10.1016/j.geoderma.2021.115212

[B19] Jiang L. Wang H. Li S. Dai X. Meng S. Fu X.. (2023). A ‘Get-Save-Return’ process continuum runs on phosphorus economy among subtropical tree species. J. Ecol. 111, 861–874. doi: 10.5061/dryad.tht76hf3c

[B20] Jiang L. Wang H. M. Li S. G. Fu X. L. Dai X. Q. Yan H.. (2021). Mycorrhizal and environmental controls over root trait–decomposition linkage of woody trees. New Phytol. 229, 284–295. doi: 10.1111/nph.16844 32761622

[B21] Killingbeck K. T. (1996). Nutrients in senesced leaves: Keys to the search for potential resorption and resorption proficiency. Ecology 77, 1716–1727. doi: 10.2307/2265777

[B22] Kong D. L. Ma C. E. Zhang Q. Li L. Chen X. Y. Zeng H.. (2014). Leading dimensions in absorptive root trait variation across 96 subtropical forest species. New Phytol. 203, 863–872. doi: 10.1111/nph.12842 24824672

[B23] Kou L. McCormack M. L. Chen W. W. Guo D. L. Wang H. M. Gao W. L.. (2017). Nitrogen ion form and spatio-temporal variation in root distribution mediate nitrogen effects on lifespan of ectomycorrhizal roots. Plant Soil. 411, 261–273. doi: 10.1007/s11104-016-3018-7

[B24] Lambers H. (2022). Phosphorus acquisition and utilization in plants. Annu. Rev. Plant Biol. 73, 17–42. doi: 10.1146/annurev-arplant-102720-125738 34910587

[B25] Lebauer D. S. Treseder K. K. (2008). Nitrogen limitation of net primary productivity in terrestrial ecosystems is globally distributed. ESA 89 (2), 371–379. doi: 10.1890/06-2057.1 18409427

[B26] Li Q. Bai W. Guo Y. Sheng J. Yuan Y. Zhang W. H.. (2023). The response of two nutrient acquisition strategies: root traits and leaf nutrient resorption and their relationships to long-term mowing in a temperate steppe. Plant Soil 491, 191–203. doi: 10.1007/s11104-022-05533-y

[B27] Li Y. Feng P. (2019). Bamboo resources in China based on the ninth national forest inventory data. World Bamboo. Ratt. 17, 45–48. doi: 10.12168/sjzttx.2019.06.010

[B28] Li L. Yu M. Yao W. Ding Y. Lin S. (2023). Research advance in growth and development of bamboo organs. Ind. Crops Prod. 205, 117428. doi: 10.1016/j.indcrop.2023.117428

[B29] Liese R. Alings K. Meier I. C. (2017). Root branching is a leading root trait of the plant economics spectrum in temperate trees. Front. Plant Sci. 8. doi: 10.3389/fpls.2017.00315 PMC5340746 28337213

[B30] Luan J. Li S. Liu S. Wang Y. Ding L. Lu H.. (2024). Biodiversity mitigates drought effects in the decomposer system across biomes. PNAS 121 (13), e2313334121. doi: 10.1073/pnas.2313334121 38498717 PMC10990129

[B31] Ma Z. Q. Guo D. L. Xu X. L. Lu M. Z. Bardgett R. D. Eissenstat D. M.. (2018). Erratum: evolutionary history resolves global organization of root functional traits. Nature 555, 94–97. doi: 10.1038/nature25783 29466331

[B32] Malchair S. Carnol M. (2009). Microbial biomass and C and N transformations in forest floors under European beech, sessile oak, Norway spruce and Douglas-fir at four temperate forest sites. Soil Biol. Biochem. 41, 831–839. doi: 10.1016/j.soilbio.2009.02.004

[B33] Mason R. E. Craine J. M. Lany N. K. Jonard M. Ollinger S. V. Groffman P. M.. (2022). Evidence, causes, and consequences of declining nitrogen availability in terrestrial ecosystems. Science 376, eabh3767. doi: 10.1126/science.abh3767 35420945

[B34] McCormack M. L. Dickie I. A. Eissenstat D. M. Fahey T. J. Fernandez C. W. Guo D.. (2015). Redefining fine roots improves understanding of below-ground contributions to terrestrial biosphere processes. New Phytol. 207, 505–518. doi: 10.1111/nph.13363 25756288

[B35] Ni H. Su W. (2024). Spatial distribution of fine root traits in relation to soil properties and aggregate stability of intensively managed Moso bamboo (Phyllostachys edulis) plantations in subtropical China. Plant Soil 498, 487–503. doi: 10.1007/s11104-023-06449-x

[B36] Ni H. Su W. Fan S. Chu H. (2021). Effects of intensive management practices on rhizosphere soil properties, root growth, and nutrient uptake in Moso bamboo plantations in subtropical China. For. Ecol. Manage. 493, 119083. doi: 10.1016/j.foreco.2021.119083

[B37] Paul M. J. Pellny T. K. (2003). Carbon metabolite feedback regulation of leaf photosynthesis and development. J. Exp. Bot. 54, 539–547. doi: 10.1093/jxb/erg052 12508065

[B38] Perakis S. S. Hedin L. O. (2002). Nitrogen loss from unpolluted South American forests mainly via dissolved organic compounds. Nature 415, 416–419. doi: 10.1038/415416a 11807551

[B39] Pregitzer K. S. DeForest J. L. Burton A. J. Allen M. F. Ruess R. W. Hendrick R. L. (2002). Fine root architecture of nine North American trees. Ecol. Monogr. 72, 293–309. doi: 10.1890/0012-9615(2002)072[0293:FRAONN]2.0.CO;2

[B40] Reich P. B. (2014). The world-wide ‘fast–slow’ plant economics spectrum: A traits manifesto. J. Ecol. 102, 275–301. doi: 10.1111/1365-2745.12211

[B41] Schachtman D. P. Reid R. J. Ayling S. M. (1998). Phosphorus uptake by plants: from soil to cell. Plant Physiol. 116, 447–453. doi: 10.1104/pp.116.2.447 9490752 PMC1539172

[B42] Shi X. Z. Yu D. S. Warner E. D. Sun W. X. Petersen G. W. Gong Z. T.. (2006). Cross-reference system for translating between genetic soil classification of China and soil taxonomy. Soil Sci. Soc Am. J. 70, 78–83. doi: 10.2136/sssaj2004.0318

[B43] Shi W. Zhu Y. Xing Y. Wu H. Ying Y. (2024). Enhancing phosphorus-solubilizing microorganism potential for alleviating plant phosphorus limitation through amino acid co-application. Appl. Soil Ecol. 204, 105714. doi: 10.1016/j.apsoil.2024.105714

[B44] Song X. Chen X. Zhou G. Jiang H. Peng C. (2017). Observed high and persistent carbon uptake by Moso bamboo forests and its response to environmental drivers. Agric. For. Meteorol. 247, 467–475. doi: 10.1016/j.agrformet.2017.09.001

[B45] Song Q. N. Ouyang M. Yang Q. P. Lu H. Yang G. Y. Chen F. S.. (2016). Degradation of litter quality and decline of soil nitrogen mineralization after moso bamboo (Phyllostachys pubscens) expansion to neighboring broadleaved forest in subtropical China. Plant Soil. 404, 113–124. doi: 10.1007/s11104-016-2835-z

[B46] Song X. Peng C. Ciais P. Li Q. Xiang W. Xiao W.. (2020). Nitrogen addition increased CO_2_ uptake more than non-CO_2_ greenhouse gases emissions in a Moso bamboo forest. Sci. Adv. 6, eaaw5790. doi: 10.1126/sciadv.aaw5790 32206705 PMC7080497

[B47] Song X. Peng C. Zhou G. Gu H. Li Q. Zhang C. (2016). Dynamic allocation and transfer of non-structural carbohydrates, a possible mechanism for the explosive growth of Moso bamboo (Phyllostachys heterocycla). Sci. Rep. 6, 25908. doi: 10.1038/srep25908 27181522 PMC4867622

[B48] Vance E. D. Brookes P. C. Jenkinson D. S. (1987). An extraction method for measuring soil microbial biomass C. Soil Biol. Biochem. 19, 703–707. doi: 10.1016/0038-0717(87)90052-6

[B49] Wang Z. Q. Gong H. Y. Sardans J. Zhou Q. P. Deng J. M. Niklas K. J.. (2022). Divergent nitrogen and phosphorus allocation strategies in terrestrial plant leaves and fine roots: A global meta-analysis. J. Ecol. 110, 2745–2758. doi: 10.1111/1365-2745.13985

[B50] Wang G. Lin G. Zhang Y. Zheng L. Zeng D. H. Lambers H. (2024). Shifts from an extensive to an intensive root nutrient-acquisition mode with stand development of three Pinus species. J. Ecol. 112, 886–900. doi: 10.1111/1365-2745.14277

[B51] Wang M. Murphy M. T. Moore T. R. (2014). Nutrient resorption of two evergreen shrubs in response to long-term fertilization in a bog. Oecologia 174, 365–377. doi: 10.1007/s00442-013-2784-7 24078082

[B52] Wang J. Shi L. Zhai L. Zhang H. Wang S. Zou J.. (2021). Analysis of the long-term effectiveness of biochar immobilization remediation on heavy metal contaminated soil and the potential environmental factors weakening the remediation effect: a review. EES 207, 111261. doi: 10.1016/j.ecoenv.2020.111261 32950873

[B53] Wen Z. Li H. Shen Q. Tang X. Xiong C. Li H.. (2019). Tradeoffs among root morphology, exudation and mycorrhizal symbioses for phosphorus-acquisition strategies of 16 crop species. New Phytol. 223, 882–895. doi: 10.1111/nph.15833 30932187

[B54] Wright I. J. Westoby I. (2003). Nutrient concentration, resorption and lifespan: Leaf traits of Australian sclerophyll species. Funct. Ecol. 17, 10–19. doi: 10.1046/j.1365-2435.2003.00694.x

[B55] Yaffar D. Cabugao K. G. Meier I. C. (2022). Representing root physiological traits in the root economic space framework. New Phytol. 234, 837–849. doi: 10.1111/nph.18070 35355283

[B56] Yan H. Freschet G. T. Wang H. M. Hogan J. A. Li S. G. Valverde-Barrants O. J.. (2022). Mycorrhizal symbiosis pathway and edaphic fertility frame root economics space among tree species. New Phytol. 234, 1639–1653. doi: 10.1111/nph.18066 35243647

[B57] Yang C. B. Ni H. J. Zhang Z. K. Zhang X. P. Bian F. Y. (2019). Changes in soil carbon pools and components induced by replacing secondary evergreen broadleaf forest with Moso bamboo plantations in subtropical China. CATENA 180, 309–319. doi: 10.1016/j.catena.2019.02.024

[B58] Yang F. Wang B. Shi Z. Li L. Li Y. Mao Z.. (2021). Immobilization of heavy metals (Cd, Zn, and Pb) in different contaminated soils with swine manure biochar. Environ. Pollut. Bioavailabil. 33, 55–65. doi: 10.1080/26395940.2021.1916407

[B59] Yang K. Zhu C. Zhang J. Li Z. Liu Y. Song X.. (2022). Nitrogen fertilization in bamboo forest accelerates the shoot growth and alters the lignification process in shoots. Ind. Crops Prod. 187, 115368. doi: 10.1016/j.indcrop.2022.115368

[B60] Yao Y. Cao S. Gong X. Singh B. P. Fang Y. Ge T.. (2022). Intensive management of a bamboo forest significantly enhanced soil nutrient concentrations but decreased soil microbial biomass and enzyme activity: a long-term chronosequence study. J. Soils. Sediments. 22, 2640–2653. doi: 10.1007/s11368-022-03253-5

[B61] Yuan Z. Chen H. (2015). Negative effects of fertilization on plant nutrient resorption. Ecol 96, 373–380. doi: 10.1890/14-0140.1 26240859

[B62] Zheng J. Freschet G. T. Tedersoo L. Li S. Yan H. Jiang L.. (2024). A trait-based root acquisition-defence-decomposition framework in angiosperm tree species. Nat. Commun. 15, 5311. doi: 10.1038/s41467-024-49666-3 38906891 PMC11192760

[B63] Zheng C. Yang Z. Si M. Zhu F. Yang W. Zhao F.. (2021). Application of biochars in the remediation of chromium contamination: Fabrication, mechanisms, and interfering species. J. Hazard. Mater. 407, 124376. doi: 10.1016/j.jhazmat.2020.124376 33144008

[B64] Zhu Y. Xing Y. Li Y. Jia J. Ying Y. Shi W. (2024). The role of phosphate-solubilizing microbial interactions in phosphorus activation and utilization in plant–soil systems: A review. Plants 13, 2686. doi: 10.3390/plants13192686 39409556 PMC11478493

[B65] Zuo K. Fan L. Guo Z. Zhang L. Duan Y. Zhang J.. (2024). High nutrient utilization and resorption efficiency promote bamboo expansion and invasion. JEM 362, 121370. doi: 10.1016/j.jenvman.2024.121370 38838536

