# Supplementary material for: Nitrogen trade-offs between roots and leaves of Moso bamboo and different effects of management practices on root traits and processes in subtropical forests
Source: Front Plant Sci. 2025 May 15;16:1583127. doi: 10.3389/fpls.2025.1583127 (PMC12124129; doi:10.3389/fpls.2025.1583127)
Supplement: Supplementary file 1 [file DataSheet1.docx]

**Supplement materials**

Nitrogen trade-offs between roots and leaves of Moso bamboo and different effects of management practices on root traits and processes in subtropical forests

Junhui Jiang^a^, Wenhui Shi^a^, Yu Fu^a^, Yuelin He^a^, Shuyang Wang^a^, Yeqing Ying^a,*^, Lei Jiang^a,*^

^a^ State Key Laboratory of Subtropical Silviculture, Zhejiang A&F University, Hangzhou, 311300, China

* Corresponding author

Tel.: +86 0571-63719032; e-mail address: yeqing@zafu.edu.cn

Tel.: +86 0571-63719032; e-mail address: jiangl1225@zafu.edu.cn


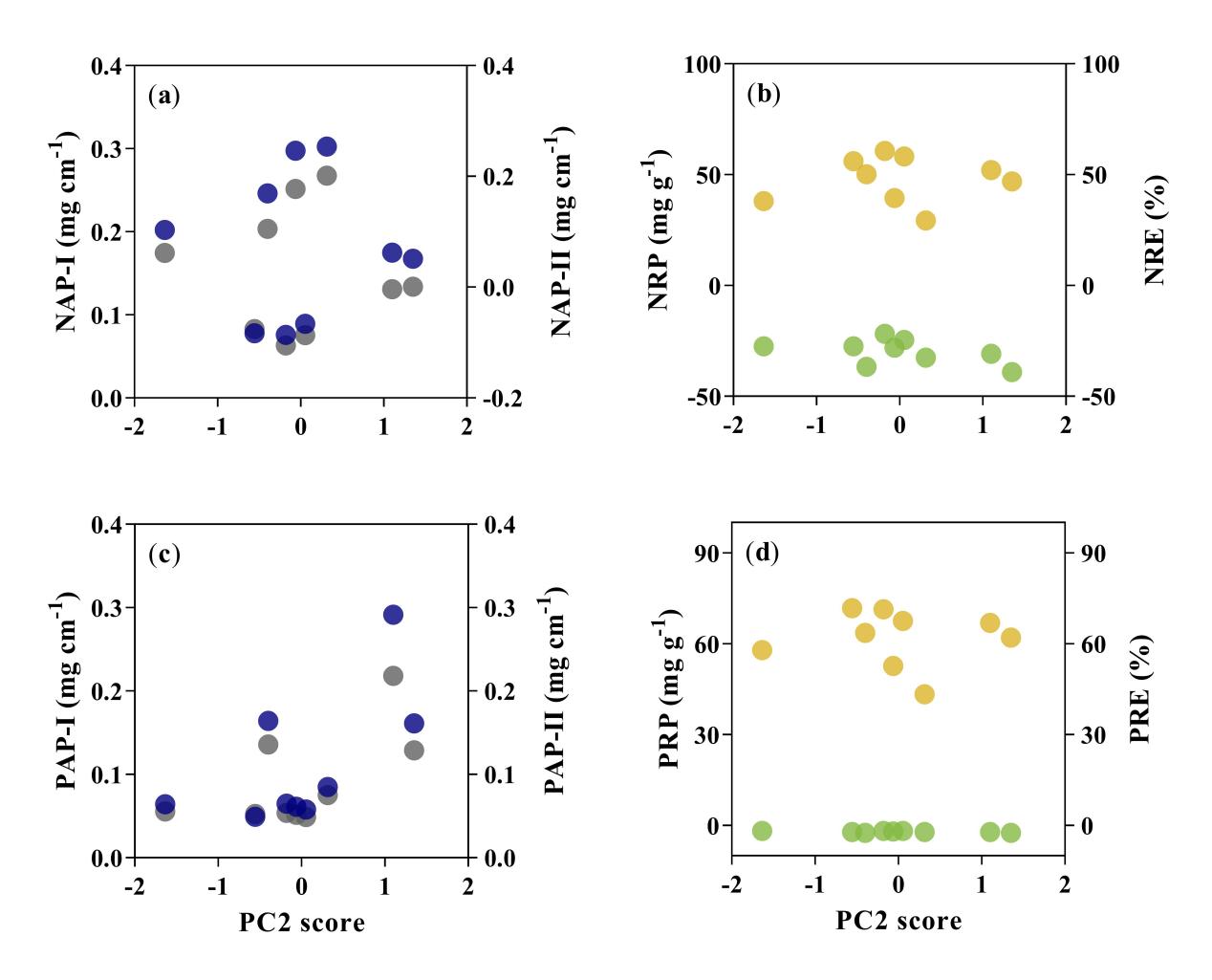


**Fig. S1** Principal component analysis (PCA) for root traits and relationships of the loading score of the second principal component analysis (PC2) with the nutrient-associated processes. Relationships of PC2 score with the nitrogen absorption potential (**a**, NAP-I (blue dots), NAP-II (gray dots), nitrogen resorption proficiency (the negative of nitrogen concentration in leaf litter was used here) and nitrogen resorption efficiency (**b**, NRP (green dots), NRE (yellow dots)), phosphorus absorption potential (**c**, PAP-I (blue dots); b, PAP-II (gray dots)), phosphorus resorption proficiency (the negative of nitrogen concentration in leaf litter was used here) and phosphorus resorption efficiency (**d**, PRP (green dots), PRE (yellow dots)).


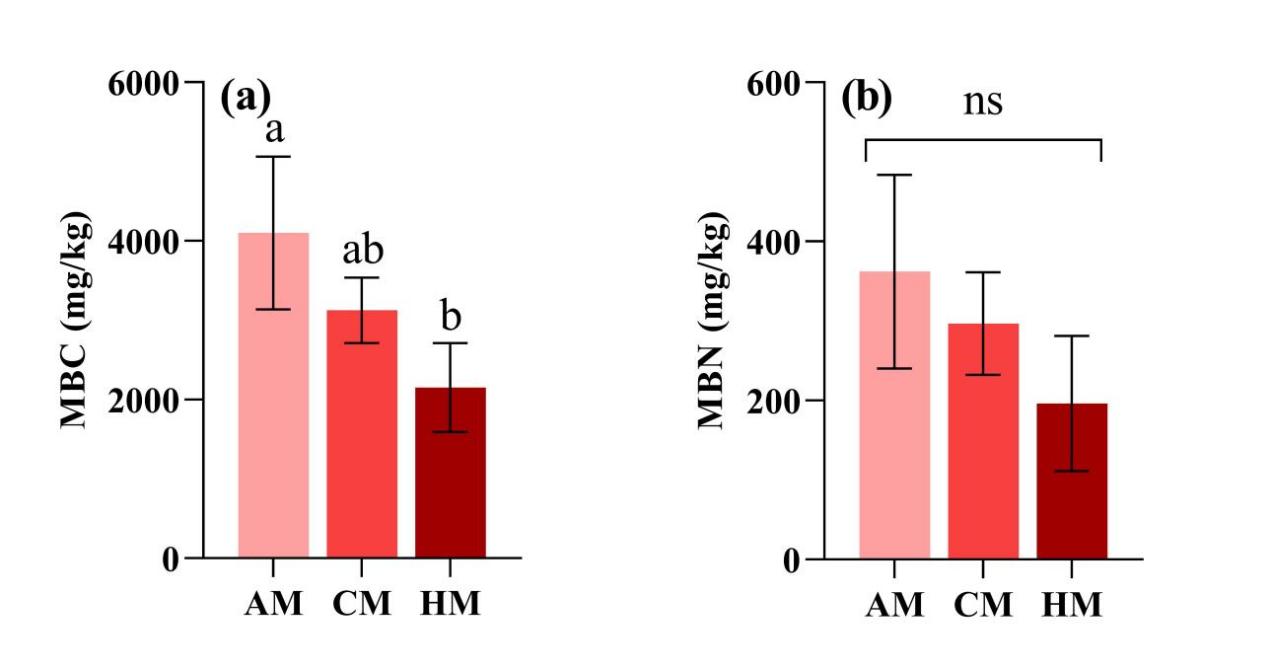


**Fig. S2** Effects of different management practices on soil microbial biomass carbon (MBC, **a**) and microbial biomass nitrogen (MCN, **b**). Values are mean ± SE (*n* = 3). Tukey's honest significant difference test was used to determine significant differences between means. Different letters indicate significant differences between treatments (*P* < 0.05).


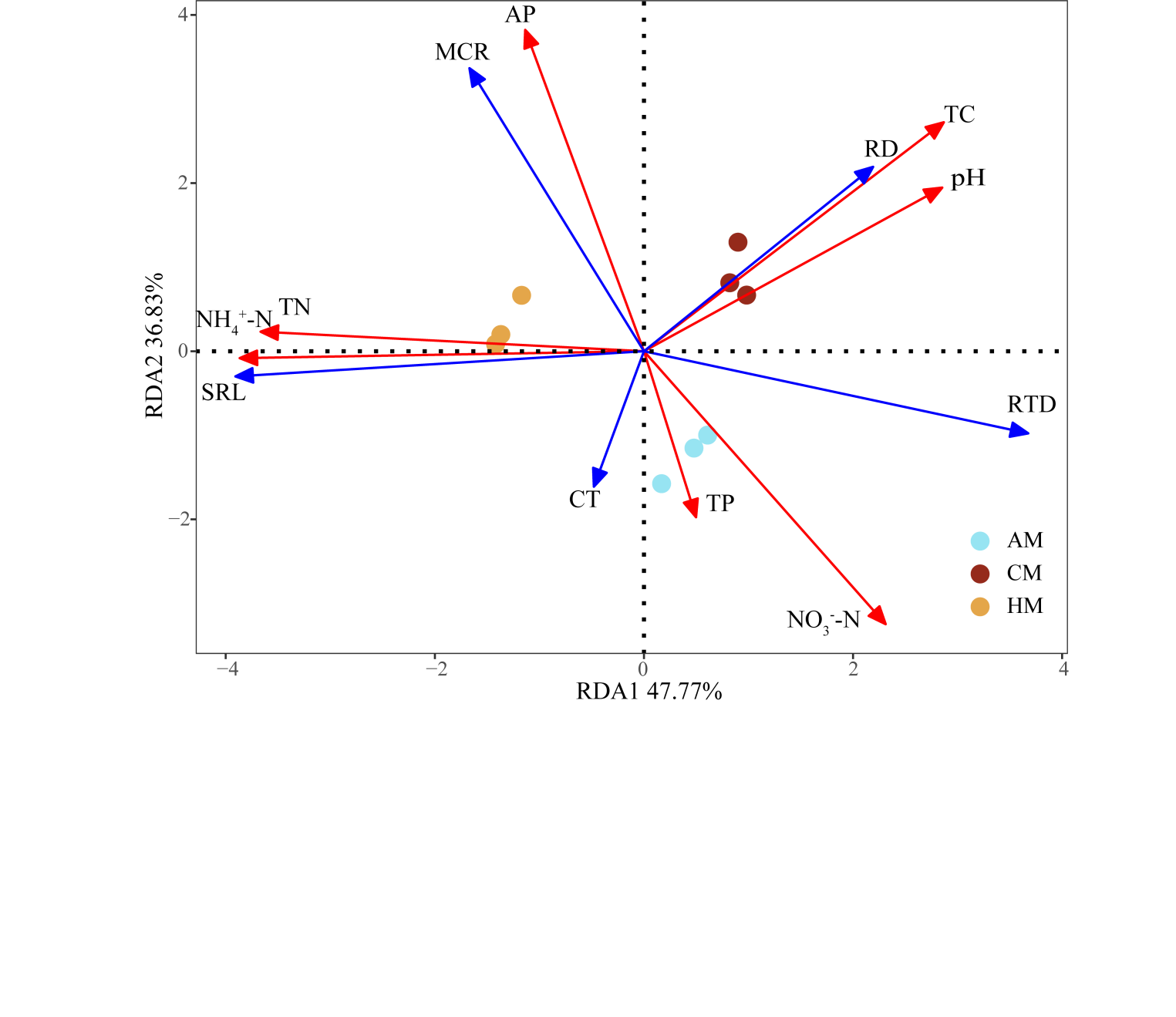


**Fig. S3** Redundancy analysis of soil nutrient content and root traits of bamboo. RD, root diameter (mm), SRL, specific root length (m g^-1^), RTD, root tissue density (g cm^-3^); BI, branching intensity (tips cm^-1^), CT, cortex thickness (mm); MCR, mycorrhizal colonization rate (%); TC, soil total carbon (mg g^-1^), TN, soil total nitrogen (mg g'); NH_4_^+^-N (mg kg^-1^), NO_3_^-^-N (mg kg^-1^), AP, available P (mg kg^-1^).

**Table S1** The background soil properties for Moso bamboo under three managements.

| Treatments | pH | Total C (mg g^-1^) | Total N (mg g^-1^) | Total P (mg g^-1^) | C/N | N/P | NH_4_^+^-N (mg kg^-1^) | NO_3_^-^-N (mg kg^-1^) | Available P (mg kg^-1^) |
| --- | --- | --- | --- | --- | --- | --- | --- | --- | --- |
| AM | 5.48 ± 0.18^ab^ | 21.83 ± 0.06^b^ | 2.17 ± 0.06^b^ | 5.63 ± 0.15^b^ | 9.99 ± 0.20^b^ | 0.39 ± 0.003^b^ | 15.54 ± 5.56^b^ | 7.41 ± 3.07^a^ | 2.33 ± 1.15^b^ |
| CM | 6.03 ± 0.18^a^ | 33.3 ± 0.17^a^ | 2.57 ± 0.06^a^ | 5.47 ± 0.15^b^ | 13.09 ± 0.16^a^ | 0.47 ± 0.001^a^ | 12.05 ± 1.50^c^ | 3.94 ± 0.34^b^ | 6.70 ± 0.53^a^ |
| HM | 5.29 ± 0.18^b^ | 20.93 ± 0.21^c^ | 2.50 ± 0.10^a^ | 6.57 ± 0.21^a^ | 8.38 ± 0.16^c^ | 0.38 ± 0.003^c^ | 25.98 ± 1.13^a^ | 2.23 ± 0.18^c^ | 6.63 ± 0.21^a^ |

Values are means and stand errors (in parentheses). Significant differences between means were determined using Tukey’s honestly significant difference test. Different letters in a column indicate significant differences between three managements (*n* = 3, *P* < 0.05). AM, abandonment managements; CM, conventional management; HM, high-intensity management.

**Table S2** Correlation analysis between soil properties and root traits of moso bamboo under three managements.

|  | NAP-I | NAP-II | PAP-I | PAP-II | NRP | PRP | NRE | PRE | RD | SRL | RTD | BI | SD | CT | MCR | MBC | MBN |
| --- | --- | --- | --- | --- | --- | --- | --- | --- | --- | --- | --- | --- | --- | --- | --- | --- | --- |
| NAP-I | 1 |  |  |  |  |  |  |  |  |  |  |  |  |  |  |  |  |
| NAP-II | 0.99^***^ | 1 |  |  |  |  |  |  |  |  |  |  |  |  |  |  |  |
| PAP-I | 0.25 | 0.16 | 1 |  |  |  |  |  |  |  |  |  |  |  |  |  |  |
| PAP-II | 0.28 | 0.19 | 1.00^***^ | 1 |  |  |  |  |  |  |  |  |  |  |  |  |  |
| NRP | 0.46 | 0.42 | 0.49 | 0.53 | 1 |  |  |  |  |  |  |  |  |  |  |  |  |
| PRP | 0.31 | 0.29 | 0.50 | 0.54 | 0.92^***^ | 1 |  |  |  |  |  |  |  |  |  |  |  |
| NRE | -0.75^*^ | -0.79^*^ | 0.24 | 0.22 | -0.41 | -0.23 | 1 |  |  |  |  |  |  |  |  |  |  |
| PRE | -0.78^*^ | -0.81^**^ | 0.14 | 0.13 | -0.33 | -0.11 | 0.97^***^ | 1 |  |  |  |  |  |  |  |  |  |
| RD | 0.39 | 0.45 | -0.37 | -0.36 | -0.07 | -0.22 | -0.53 | -0.49 | 1 |  |  |  |  |  |  |  |  |
| SRL | -0.74^*^ | -0.71^*^ | -0.26 | -0.26 | -0.42 | -0.13 | 0.63 | 0.69^*^ | -0.64 | 1 |  |  |  |  |  |  |  |
| RTD | 0.68^*^ | 0.62 | 0.56 | 0.54 | 0.59 | 0.35 | -0.48 | -0.56 | 0.23 | -0.88^**^ | 1 |  |  |  |  |  |  |
| BI | 0.10 | 0.06 | 0.36 | 0.33 | 0.58 | 0.48 | -0.33 | -0.36 | -0.35 | -0.20 | 0.51 | 1 |  |  |  |  |  |
| SD | 0.18 | 0.22 | 0.47 | 0.39 | -0.58 | -0.30 | -0.20 | -0.25 | -0.18 | -0.62 | 0.83^**^ | 0.62 | 1 |  |  |  |  |
| CT | 0.42 | 0.42 | 0.44 | 0.47 | 0.24 | 0.20 | -0.12 | -0.19 | 0.14 | -0.31 | 0.31 | 0.03 | 0.30 | 1 |  |  |  |
| MCR | -0.23 | -0.17 | -0.79* | -0.84 | 0.83 | 0.79 | -0.06 | -0.08 | 0.46 | 0.32 | -0.58 | -0.45 | -0.56 | -0.28 | 1 |  |  |
| MBC | 0.21 | 0.15 | 0.43 | 0.42 | 0.75^*^ | 0.62 | -0.21 | -0.16 | 0.01 | -0.49 | 0.64 | 0.52 | 0.54 | -0.28 | -0.76^*^ | 1 |  |
| MBN | 0.09 | 0.09 | -0.27 | -0.27 | 0.41 | 0.27 | -0.38 | -0.26 | 0.25 | -0.30 | 0.22 | 0.17 | 0.03 | -0.67^*^ | -0.19 | 0.73^*^ | 1 |

Asterisks indicate a significant relationship (**P* < 0.05, ***P* < 0.01, ****P* < 0.001). NAP-I and NAP-II are the nitrogen absorption potential, PAP-I and PAP-II are the phosphorus absorption potential, NRP is the nitrogen resorption proficiency (the negative of nitrogen concentration in leaf litter was used here), NRE is the nitrogen resorption efficiency, PRP is the phosphorus resorption proficiency (the negative of phosphorus concentration in leaf litter was used here), and PRE is the phosphorus resorption efficiency. RD, root diameter (mm); SRL, specific root length (m g^-1^); RTD, root tissue density (g cm^-3^); BI, branching intensity (tips cm-1); Stele diameter (SD, mm); CT, cortex thickness (mm); MCR, mycorrhizal colonization rate (%); MBC, microbial biomass carbon (mg kg^-1^); MCN, microbial biomass nitrogen (mg kg^-1^).
